# Assessment of prices, availability and affordability of essential medicines in Juba County, South Sudan

**DOI:** 10.1186/s40545-023-00675-5

**Published:** 2023-12-30

**Authors:** Justin Deng, Augustino Ting Mayai, Egide Kayitare, Theoneste Ntakirutimana, Omary Swallehe, Thomas Bizimana

**Affiliations:** 1 EAC Regional Centre of Excellence for Vaccines, Immunization, and Health Supply Chain Management, College of Medicine and Health Sciences, University of Rwanda, Kigali, Rwanda; 2 Department of Pharmacy, School of Medicine and Pharmacy, College of Medicine and Health Sciences (CMHS), University of Rwanda, Kigali, Rwanda; 3 Sudd Institute, Juba, South Sudan; 4 Department of Environmental Health Sciences, School of Public Health, College of Medicine and Health Sciences (CMHS), University of Rwanda, Kigali, Rwanda; 5 Department of Business Studies, School of Business, Dar es Salaam Campus College, Mzumbe University, Mzumbe, Tanzania

**Keywords:** Lowest priced generic, Originator brand, Availability, Affordability, Accessibility, Prices, Tracer medicines, South Sudan

## Abstract

**Background:**

Access to safe, effective, affordable, and high-quality medications has been included in the Sustainable Development Goals (SDGs) of the United Nations as a crucial step towards attaining universal health coverage. Access to medicines is a fundamental human right. If medicines are accessible and affordable, they save lives by reducing mortality and morbidity associated with acute and chronic diseases. WHO recommends that all countries voluntarily reach the minimum target of 80% availability of medicines by 2025. The primary purpose of this research is to assess access to essential medicines in Juba County, South Sudan.

**Methods:**

This study was undertaken using the standard World Health Organization/Health Action International Organization (WHO/HAI) approach for surveying the prices, availability, and affordability of medicines. A survey was conducted in six payams of Juba County, South Sudan, and 55 health facilities were assessed.

**Results:**

Prices for generic medicines were better in faith-based health facilities with a median price ratio of 1.95. Private pharmacies and private clinics had MPRs of 4.64 and 4.32, respectively. Local prices were high compared to International referent prices. Availability of medicines was highest in the faith-based health facilities (65.5%) and slightly lower in private pharmacies (55.4%), private clinics (57.7%) and public (50.4%) sectors. Most of the surveyed medicines were unaffordable. The medicines needed to treat non-communicable diseases cost up to 33.7-day wages for one full course of treatment.

**Conclusions:**

In South Sudan, medicines are poorly available in all sectors. Medicines are affordable in the public sector but Most medicines are unaffordable in private pharmacies, private clinics and faith-based health facilities. Poor medicines availability in the public sector contributes to the overall unaffordability of medicines in all the other sectors.

## Background

Medicines are essential in health care. If they are accessible and affordable, they save lives by reducing mortality and morbidity associated with acute and chronic diseases. Thus, medicines’ access is a fundamental human right. However, access to medicines is hampered by several factors, such as low availability and low affordability. These three factors prevent a large portion of the population from accessing medicines, which might have negative health implications for patients [1]

Among the key policy goals of Countries is the national drug policy to ensure the availability and affordability of essential medicines. To achieve the stated objectives, low-and-middle-income countries compile life-saving medicines into national essential medicines lists (NEMLs) [2].

Access to safe, effective, quality, and affordable medicines has been included in the United Nations’ Sustainable Development Goals (SDGs) as a critical step towards universal health coverage [3].

WHO recommends the minimum target of 80% availability of affordable, safe and quality essential medicines and basic health technologies to be voluntarily attained by all the countries by 2025. However, in low-and-middle-income countries (LMICs), meeting this target is still a major challenge as studies report low availability and poor affordability [4, 5]

The World Health Organization report showed that More than 30% of the world’s population does not have consistent access to essential medicines. In some of Africa’s and Asia’s poorest nations, this number may reach 50%. In underdeveloped countries, poor medicines availability, high medicine prices, and low affordability are major barriers to essential medicines access [1, 2, 6]

According to a survey done in 36 nations, the average availability of essential medicines in the public and private sectors was 38% and 64%, respectively [7, 8]. Babar and colleagues carried out a study in 52 LMICs on three asthma medicines (budesonide beclomethasone and salbutamol) and discovered poor availability and affordability of these medicines across many African countries, including Malawi, Egypt and Burundi [4].

Prices, availability, and affordability of essential medicines have been studied in many low-income countries; however, there is currently little information available concerning South Sudan. The health system of South Sudan remains fragile after decades of regional and internal conflict. The essential medicines needed to combat the risks associated with this fragility are routinely unavailable at county health departments.

South Sudan has been working towards improving its healthcare system since its independence. However, this effort has been hindered by many challenges, such as prolonged conflict, poverty, poor pharmaceutical supply management practices, inadequately skilled workforce, lack of adequate infrastructure, and the worsening impacts of climate change resulting in plodding progress over the last couple of years [9].

The South Sudan MOH, USAID, HPF and World Bank have implemented the Emergency Medicines Fund (EMF) to continue improving the provision of essential medicines and quality health services.

Despite this effort exerted by South Sudan and its partners, the country still faces challenges in accessing medicines [9]. when medicines are not available in public health facilities, patients are forced to buy them from private pharmacies without being reimbursed, except for some of the few people who are covered by private health insurance.

In South Sudan, few people have health insurance, making it difficult for many to pay medical bills. South Sudan's health system faces many funding challenges contributing to shortages of essential medicines in public health facilities. In addition, there is a limitation of data on prices and availability of essential medicines, making it difficult for policymakers to make decisions on access to essential medicines. Thus, studies are needed to generate data to make decisions on the prices, availability, and affordability of essential medicines.

Therefore, this study aimed to determine essential medicines’ prices, availability, and affordability in Juba County, South Sudan. The findings of this study would help to measure South Sudan's progress in achieving the goal of increasing the availability and affordability of medicines. Twenty-nine essential medicines (15 tracer medicines from South Sudan and 14 from the WHO core list) were investigated**.**

Tracer medicines are a subset of medicines chosen by the South Sudanese government to monitor the availability of medicines in the public sector. The availability of tracer medicines in public health facilities implies that the community is receiving basic health care. In contrast, the unavailability of tracer medicines indicates the deterioration of the pharmaceutical supply chain. The scarcity of tracer medicines jeopardizes the fulfilment of UN Sustainable Development Goal 3 on health [3].

## Methods

The survey used the Standardized methodology of the World Health Organization and International Health Action Organization (WHO/HAI) for a survey of prices, availability and affordability of medicines [10].

### Study design

This study used a descriptive cross-sectional research design with a quantitative approach.

### Areas surveyed

According to the standard survey method for drug accessibility research developed by the World Health Organization (WHO) and Health Action International (HAI), the scope of this survey must first be determined. This means that a survey can be conducted nationwide or regionally. For countries with vast areas of land or large populations, it is recommended that surveys can be based on regions. Considering the size and administrative divisions of Juba County, this survey selected five peripheral payams and one municipal Payam that hosts Juba City. This made up a total of six payams (administrative areas), namely: Kator payam, Rejaf payam, Juba payam, Munuki payam, Luri payam and Mangalla payam. A Payam is a second-lower administrative division in the South Sudan administrative categories below the county. All the selected six payams are reachable in a 1-day drive as per the recommendation from the WHO/HAI manual [10].

Juba County is located in Central Equatoria state and is the largest county among the 79 counties of South Sudan. The estimated population was 499, 538 by 2020. It hosts the capital city of South Sudan and has become a multi-ethnic centre. The county has 17 payams in which many health facilities are located.

### Sectors and health facilities surveyed

Prices and availability of medicines were recorded from 55 health facilities comprising 15 Public health facilities, 18 private pharmacies, 18 private clinics and 4 faith-based health facilities across 6 Payams of Juba County. In addition, the central procurement data were obtained from HPF national office (grown agent) as it purchases medicines for the government of South Sudan. This collection of central procurement data is recommended by WHO/HAI methodology.

Initially, a sample size of 58 health facilities was drawn using the WHO/HAI sampling technique from the list of 365 active health facilities (from all four sectors) obtained from the county health department. From the selected 58 facilities, 3 public health facilities were excluded, because they did not stock the survey medicines as they were specialized centres for TB, HIV/AIDS and nutrition programs; therefore, data were collected from 55 health facilities only. The surveyed health facilities comprise 6 hospitals and 49 primary health care centres (PHCC) (Table 1).

**Table 1:** Distribution of the surveyed areas, health facilities and sectors

Payam	Public facilities		Private pharmacies	Private clinics		Faith-based facilities		Total
	Hospital	Health centre	Retail pharmacies	Hospital	Health centre	Hospital	Health centre	
1. Juba*	2	0	3	1	2	0	1	9
2. Kator	0	3	3	0	3	0	1	10
3. Munuki	0	3	3	2	1	1	0	10
4. Rejaf	0	3	3	0	3	0	1	10
5. Luri	0	1	3	0	3	0	0	7
6. Mangalla	0	3	3	0	3	0	0	9
Sub-total	2	13	18	3	15	1	3	55
	15		18	18		4		

* = main urban area

### Sample size

The sample size of this study was calculated using the WHO/HAI methodology which recommends that a main public medical outlet is purposefully chosen as the starting point and the rest of the medical outlets are selected based on their proximity to the main medical outlets. Using this WHO/HAI methodology for the selection of survey institutions, a total of fifty-eight (58) medical outlets (18 public outlets, 18 private pharmacies, 18 private clinics and 4 faith-based facilities) were selected for this study. All 58 medical institutions were selected in the areas within 3-h drive from the main health facility to comply with the recommendations of the methodology. This sample size was representative considering the fewer number of public medical facilities in Juba County. From the selected 58 facilities, 3 public health facilities were excluded, because they did not stock the survey medicines as they were specialized centres for TB, HIV/AIDS and nutrition programs; therefore, data were collected from 55 facilities only.

### Sampling procedures

One major public facility was selected in each survey area and two other public medical institutions were randomly selected within a 3-h driving radius from the six public facilities that were initially selected in the first stage. Based on this criterion, a total number of 18 public medical facilities were selected in six payams. 18 retail pharmacies, 18 private clinics and 4 faith-based facilities were selected based on their proximity to the pre-selected public medical facilities. Therefore, this study intended to survey a total number of 58 different medical outlets sampled from four different sectors. The technique used here adapts the sampling procedures provided by the WHO/HAI methodology that involves many sequential steps. The first step of this sampling technique required that the main public medical outlet in each of the six study areas (designated Payams in this research) was chosen as a reference and then select the other 5 nearest public medical outs in each of the study areas. However, this procedure was not fully followed in this study, because there were fewer public medicine outlets in Juba County. Therefore, in this adapted Methodology, two public facilities nearest to the main medicine outlet were selected in addition to 1 main public facility to make three. This constitutes a total of eighteen selected public medical outlets in each of the six payams. Three private pharmacies and three private clinics were selected based on their proximity to the pre-selected main public health facility in all the six payams. If two or more private medical outlets had similar distances from the pre-selected main public medical outlet, then the survey outlets were selected using the simple random technique. Only four faith-based health facilities in Juba County were included in the sample for the survey.

### Medicines selection criteria

Medicine selection was based on the updated WHO/HAI core List and the South Sudan tracer medicines list. Only 11 of the 14 medicines on the WHO/HAI core list and 15 tracer medicines were included in the study. Three medicines (simvastatin 20 mg, bisoprolol 5 mg and captopril 25 mg tab.) from the WHO/HAI core list were excluded, because they were not on the updated South Sudan Essential Medicines List (SSEML). They were replaced by three other therapeutically equivalent medicines (atorvastatin 40 mg, propranolol 40 mg and lisinopril 10 mg) on SSEML. All the medicines included in this study had international reference prices (IRPs) in the Medicines Price Indicator Guide provided by the Management Sciences for Health (MSH) version of 2015 and were authorized for sale in the Republic of South Sudan. The dosage forms, package size, and treatment regimens were confirmed in the South Sudan essential medicines list and standard treatment guidelines in collaboration with healthcare providers. Hence, this study collected data on 29 medicines drawn from the WHO/HAI core list and tracer medicines list of South Sudan (Table 2). These medicines were used at the hospital level (Level 3). However, 27 were used at the primary healthcare centre (Level 2), and 22 were used in the primary healthcare unit (Level 1).

**Table 2:** List of medicines that were surveyed

S/no	Surveyed medicines	Daily dose (units or ml)	Included in WHO/HAI global core list	Availability on SSEML	Originator brand registered in South Sudan	Treatment duration [days]	Number of units or ml for one course of treatment
1	Albendazole (chewable) 400 mg	1	Yes	Yes	No	7	7
2	Amoxicillin 500 mg	3	Yes	Yes	No	7	21
3	Amoxicillin capsule 250 mg	3	No	Yes	No	7	21
4	Artesunate + Amodiaquine (Adult) 100 mg + 270 mg	2	No	Yes	No	3	6
5	Artesunate + Amodiaquine (Infant) 25 mg + 67.5 mg	1	No	Yes	No	3	6
6	Azithromycin 250 mg	2	No	Yes	No	3	6
7	Cefixime 20 mg/ml	5 ml	No	Yes	No	5	25 ml
8	Ceftriaxone injection 1 g/vial	1	Yes	Yes	No	1	1
9	Ciprofloxacin 500 ng	2	Yes	Yes	No	7	14
10	Co-trimoxazole suspension 8 + 40 mg	10 ml	Yes	Yes	No	7	70 ml
11	Doxycycline 100 mg	1	No	Yes	No	30	30
12	Metronidazole 200 mg	6	No	Yes	No	7	42
13	Sulfamethoxazole/Trimethoprim (Co-trimoxazole) tab 400 mg + 80 mg	3	No	Yes	No	7	21
14	Sulphadoxine + pyrimethamine 500/25 mg	1	No	Yes	No	1	1
15	Oral Rehydration Salt (ORS), WHO formulation	2	No	Yes	No	2	2
16	Zinc sulphate 20 mg	2	No	Yes	No	7	14
17	Amitriptyline 25 mg	3	Yes	Yes	No	30	90
18	Atorvastatin 40 mg	1	No	Yes	No	30	30
19	Diazepam 5 mg	1	Yes	Yes	No	30	60
20	Diclofenac 50 mg	2	Yes	Yes	No	30	60
21	Ferrous sulphate + Folic acid 200 mg + 0.25 mg	1	No	Yes	No	30	30
22	lisinopril 10 mg	1	No	Yes	No	30	30
23	Metformin 500 mg	3	Yes	Yes	No	30	90
24	Omeprazole 20 mg	1	Yes	Yes	No	30	30
25	Propranolol 40 mg	2	No	Yes	No	30	60
26	Salbutamol inhaler 100mcg/dose	as needed	No	Yes	Yes	As needed	200 doses
27	Vitamin A (Retinol) 200, 000 IU (60 mg)	1	No	Yes	No	30	30
28	Ibuprofen 400 mg	as needed	No	Yes	No	As needed	400 mg
29	Paracetamol suspension 24 mg/ml	15 ml	Yes	Yes	No	3	45 ml

### Data collection, entry and analysis

Data were collected using the data collection forms that were automatically generated from pre-programmed Excel workbooks obtained from the WHO/HAI website (https:/haiweb.org) after the entry and update of the core list of medicines provided by WHO and the local list of tracer medicines. Twelve data collectors were recruited and trained for 2 days. After the training was concluded, they were dispatched for data collection in six teams. Each team of two people was tasked to collect data in each payam. The data collection commenced on 10/03/2023 and was finished on 16/10/2023. The data collectors collected data in all four health sectors under the close supervision of the researcher. The sources of data were heads of departments in health facilities who consented to provide information about the availability of medicines and prices. Some of the challenges encountered during data collection include refusal to sign the consent form, absent of heads of departments, and difficulties in unit price calculation by data collectors, among others.

Data from the surveys were entered into the pre-programmed MS Excel Workbook provided as part of the WHO/HAI methodology. The Workbook's 'double entry' and 'data checker' functions were used to validate data entry. Errors and potential outliers were carefully verified and corrected.

Data analysis was carried out using the default settings of the automated Microsoft Excel worksheet created by WHO and HAI [10] which generated summary findings, such as percent availability, median price ratios, and cost for one treatment course. Further analysis was done to create graphs and pie charts using Microsoft Excel version 2010.

### Measurement of availability of medicines

Based on the WHO/HAI methodology, individual medicine availability was determined by the physical presence of that medicine in medicine outlets at the time of data collection. As a result, availability was calculated and reported as a percentage (%) of sampled medicine outlets per sector, where the surveyed medications were found on the day of data collection [10].

### Measurement of affordability of medicines

Medicine affordability was determined using the median patient prices of originator brands and the lowest priced generics of each medicine in local currency for a standard treatment regimen. As per the WHO and HAI methodology, affordability was expressed as the number of days' wages needed by the lowest paid unskilled government worker (LPGW) to purchase 30-day supply of the medicine to treat non-communicable diseases (NCDs) and 7-day supply of medicines to treat communicable diseases. If the cost of the full regimen does not exceed 1-day wage, then that medicine is considered affordable. The affordability was not assessed in health facilities, where medicines were not available and in public facilities, where medicines were provided free of charge. Any treatment course that requires more than 1-day wages is considered unaffordable by WHO and HAI. According to the information from the Ministry of Labor during the time of the survey, 1-day wage of the lowest paid unskilled government worker in South Sudan was 266.6667 SSP (equivalent to 0.3252 US$). The exchange rate in the Central Bank of South Sudan was 1 US $ = 820 SSP on April 2023. For medicines from the WHO and HAI global core list, the number of units for a course of treatment is defined in the WHO and HAI manual [10]. For the tracer medicines on the supplementary list, the South Sudan Standard Treatment Guideline was used to define each medicine's daily dose and treatment duration [11].

### Prices assessment

The prices were evaluated using the international reference prices (IRPs) in the 2015 Management Sciences for Health (MSH) reference. IRPs are prices offered to international non-profit organizations for the purchase of generic medications. Medicine prices were recorded in local currency (South Sudanese pound, SSP) and they were automatically converted to US $ using the exchange rate from the Central Bank of South Sudan which was (1 US $ = 820 SSP), during the month of April 2023 in which the data were collected.

The following formula was used to express median local prices as ratios to international reference prices:

$$\mathrm{Median}\;\mathrm{Price}\;\mathrm{Ratio}\left(\mathrm{MPR}\right)=\frac{Median\;local\;unit\;price}{International\;reference\;unit\;price}$$

MPR is an important indicator for assessing the availability of essential medicines, as well as the price level and international reference level of medicines in the survey area [12]. According to the WHO, an MPR of 2.5 is considered a high and excessive local price. This means that any MPR equal to or greater than 2.5 indicated that essential medicines were not affordable in Juba County.

## Results

### Medicines availability assessment

**Fig. 1:**
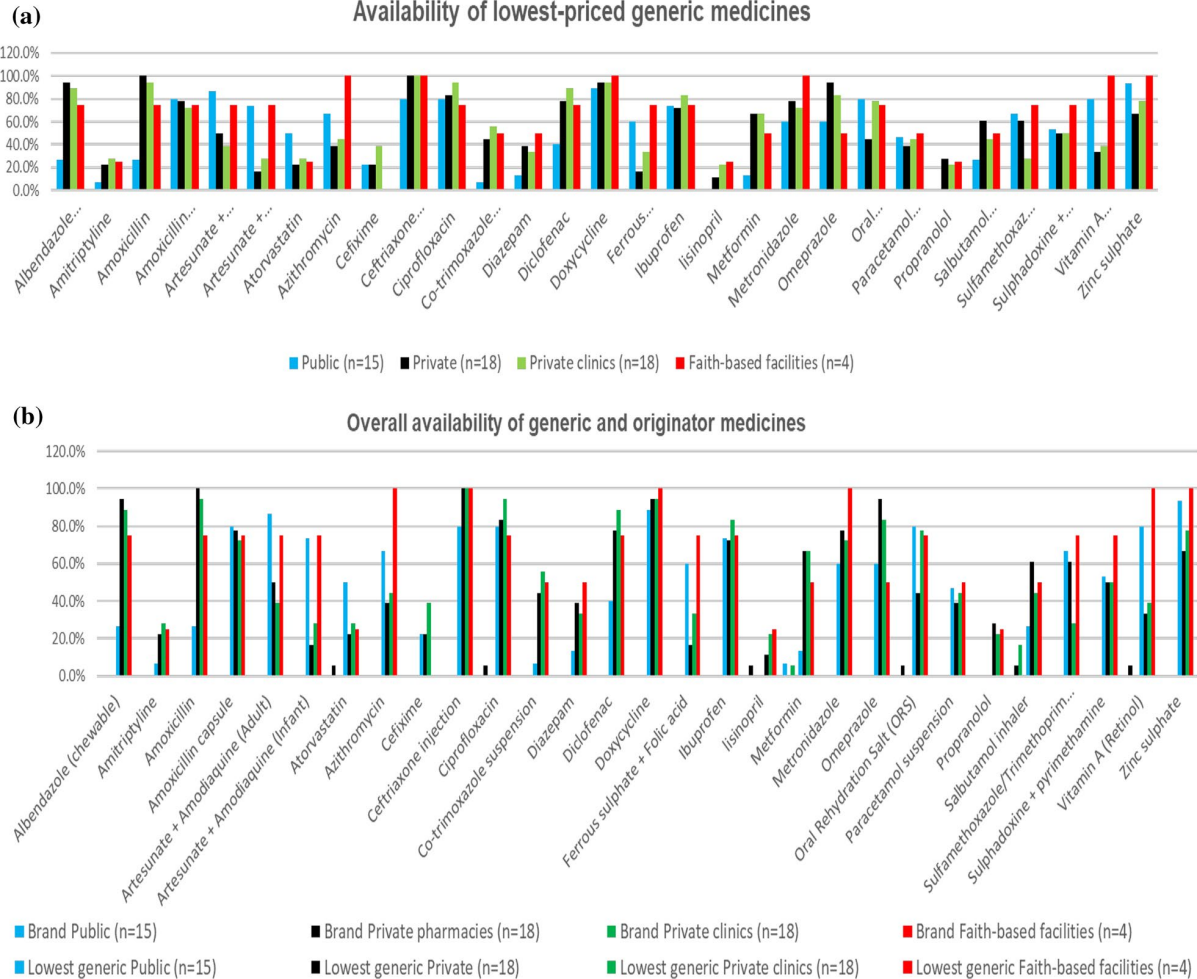
Availability of the investigated medicines in public health facilities, private pharmacies, private clinics and faith-based health facilities, **a** Availability of generic medicines. **b** Overall availability of the medicines (generic and originator brand medicines)

Generic medicines were, on average available in 55.4% of private pharmacies, 57.7% of private clinics, 65.5% of faith-based health facilities, and 50.4% of public health facilities (Fig. 1A). A few generic medicines scored 100% availability in faith-based facilities (azithromycin, ceftriaxone injection, doxycycline, metronidazole, vitamin A and zinc sulphate), and private pharmacies (amoxicillin 500 mg and ceftriaxone injection). Ceftriaxone injection, a life-saving antibiotic, was 100% available in all sectors except public health facilities, where it was found in 80% of facilities. Figure 1B depicts the combined availability of generic and originator brand medicines for every sector. The availability of all analyzed medications was moderately higher (58.4%) in private clinics and slightly lower (56.5%) and (50.6%) in private pharmacies and public facilities.

**Fig. 2:**
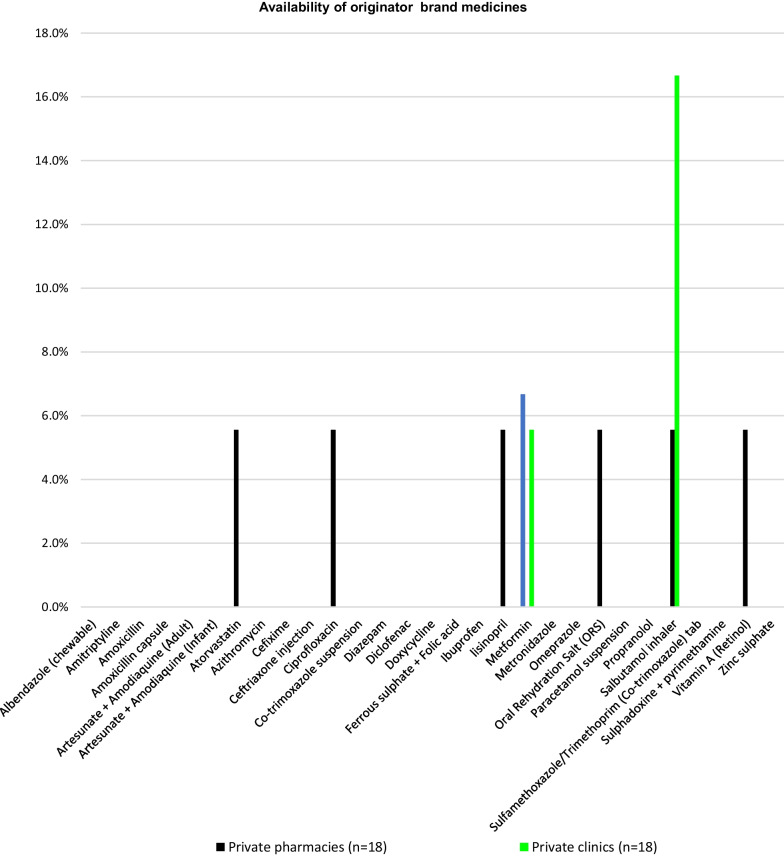
Availability of brand medicines in all the surveyed sectors

The availability of originator brand medicines was substantially low in all the surveyed sectors. Only private clinics had stocked 0.8% of brand medicines on average(Fig. 2). None of the surveyed faith-based health facilities was found to have brand medicines.\

Among the medicine groups studied, pediatric antidiarrheals and antibiotics had the highest availability. Antibiotic availability was 75% in faith-based facilities, 65.5% in private pharmacies, 64.3% in private clinics, and 58.4% in the public sector, on average. Medications for NCDs recorded the lowest availability in all the sectors.

### Medicines prices assessment

Both private pharmacies (MPR4.64) and private clinics (MPR4.32) had extremely high median MPRs. The median MPR for faith-based facilities was 1.95, about double the international reference prices. The median MPR for all generic medications purchased by the government was 1.37. The procurement prices for generic medicines were 37% higher than the international reference prices.

**Fig. 3:**
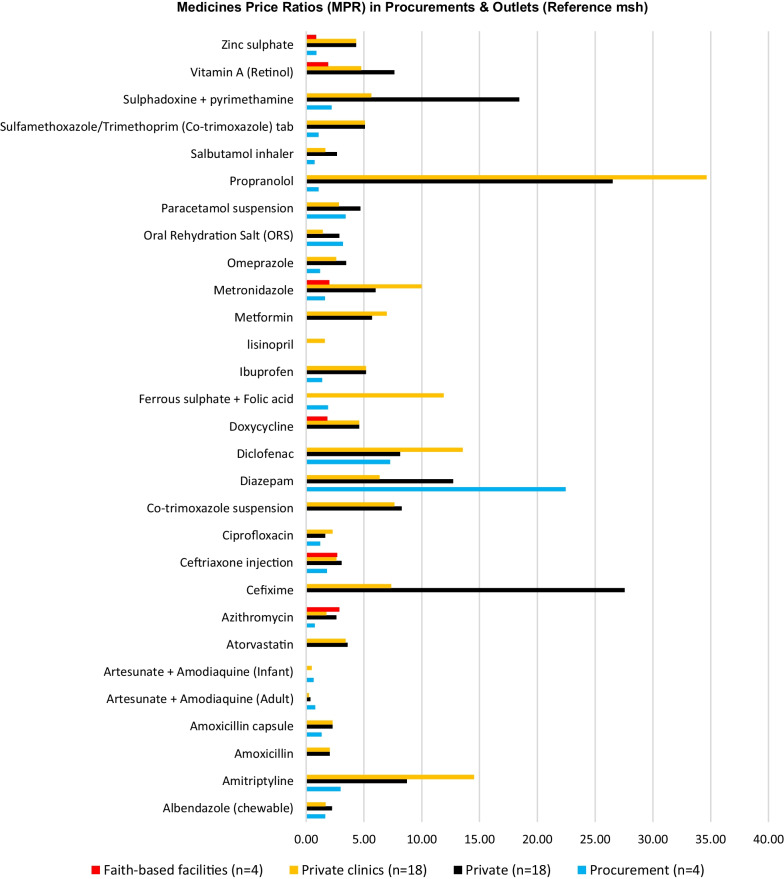
Medicines Median Price Ratios (MPR) in Procurement and Outlets

Both concentrations of artesunate tablets that were investigated showed lower median MPRs. Artesunate for adults showed MPRs of 0.24, 0.36 and 0.78 in private clinics, private pharmacies and procurement prices, respectively. In contrast, the artesunate for Pediatrics showed MPRs of 0.63 and 0.48 in procurement prices and private clinics (Fig. 3).

Other three medicines among the studied 29 medications scored low MPRs in procurement prices. These are the Azithromycin tab (MPR 0.73), salbutamol inhaler (MPR 0.73) and zinc sulphate (MPR 0.88).

None of the investigated brand medicines met the required four prices per medicine for the calculation of an MPR. Hence, No MPR was observed for brand medicines.

Due to the scarcity of originator brand medicines, a paired comparison of patient pricing for the lowest priced generics and originator brands in each sector was not possible.

Cross-sector price comparisons based on a paired analysis of lowest priced generics:

- Patient prices in private pharmacies were 215% higher than the procurement prices (19 medicines);
- Patient prices in faith-based facilities were 86.7% higher than the procurement prices (4 medicines);
- Patient prices in faith-based facilities were 56.2% lower than in private pharmacies (6 medicines); similar numbers were obtained when faith-based facilities were compared with private clinics.
- Patient prices in private clinics were 106.4% higher than the procurement prices (21 medicines); and
- Patient prices in private clinics were 3.9% lower than the private pharmacies (26 medicines);
- Patient prices in the public health facilities were not determined, because medicines were served free of charge.

MPRs for the lowest priced generics used to treat NCDs were marginally higher in private clinics and pharmacies than for antibiotics and other groups of examined medications. Furthermore, MPRs for generic drugs used to treat pain or inflammation were more elevated in private clinics than the antibiotics.

Propranolol, cefixime, Sulphadoxine + pyrimethamine and amitriptyline recorded very high MPRs with propranolol scoring an MPR of 34.64 in private clinics. Diazepam showed an abnormally high MPR (22.45) in procurement prices.

### Medicines affordability assessment

As explained in the Methods section, affordability was expressed as the number of days’ wages of the lowest paid unskilled government worker required to purchase a course of treatment based on standard treatment regimens [10]. For chronic diseases, the cost for 30 days of treatment was used in this calculation and 7 days of treatment for acute infections. Treatments are considered unaffordable if they cost more than 1-day wage, according to WHO/HAI.

**Fig. 4:**
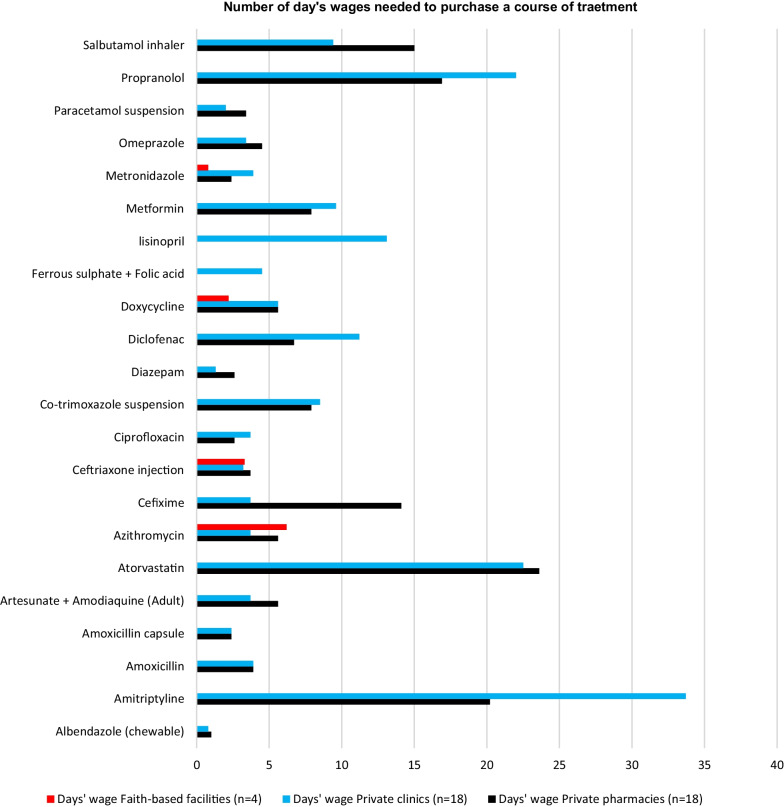
Number of day's wages required to purchase a complete course of treatment

The treatment regimen for albendazole lasted for 7 days, and it was affordable in private clinics and private pharmacies, with wages of 0.8 and 1 day. Metronidazole showed 0.8-day wage in faith-based facilities (Fig. 4).

Eight of the ten generic antibiotics assessed required more than a day's wage to purchase in private pharmacies and clinics. Cefixime was the most unaffordable antibiotic, with a single course of treatment costing up to 14-day wages in private pharmacies. All of the life-saving antibiotics were prohibitively unaffordable.

In general, NCDs needed more days' wages for a single course of treatment than the other types of medicines studied. In private clinics, amitriptyline, atorvastatin, and propranolol required 33.7-, 23-, and 22-day wages, respectively, for one complete course of treatment. The same three medications required 20.2-, 23.6- and 16.9-day wages to buy one course of treatment in private pharmacies.

## Discussion

The WHO recommends that all nations should voluntarily reach the minimum target of 80% availability of affordable, safe, and quality essential medicines and basic health technology by 2025 in order for each nation to achieve SDG 3 and UHC in respective settings [3]. Meeting this aim, however, remains a significant problem in low- and middle-income nations as findings are indicating inadequate availability and poor affordability. The findings of this study on prices, availability, and affordability of tracer medicines help to gauge South Sudan's progress in achieving this objective.

The median public procurement prices for five of the 29 generic medicines were lower than the international supplier prices included in the 2015 MSH reference prices. However, the average median MPR for public procurement prices remained higher than 1. This suggests that while public procurement was effective for these five medications, the entire procurement system remained inefficient [13].

Patient prices in private pharmacies and clinics were inconsistent and remarkably high; for example, the patient price for zinc sulphate tablets 20 mg was 5 times its government procurement price, and propranolol was more than 24 times its government procurement price. These higher prices in the private sector are attributed to custom clearance, transportation, rent, operation cost, poor regulation, and other price components. According to this survey, patients pay more for their medications at private pharmacies and clinics than in faith-based facilities. This is consistent with the majority of research conducted in LMICs, which discovered that prices in the private sector were higher than those in the public and faith-based sectors [4, 5].

According to WHO, critical medicines for NCDs should be available in at least 80%. However, the availability of the most critical drugs across all sectors in South Sudan was found to fall short of the WHO criteria in this study.

The limited availability of medicines is widespread in many LMICs. A survey conducted in 36 countries found that the average availability of essential medicines in the public and private sectors was 38% and 64%, respectively [2]. This investigation discovered that the availability of medicines in the public sector in South Sudan was higher than the reported 38%. However, availability at private pharmacies was lower than 64%. Furthermore, research in Ethiopia [14] and Jordan [7] revealed significantly higher levels of availability in both sectors than in South Sudan.

The findings of this research are a little bit higher than the average availability in Kenya [15] and Eswatini [16] which were 43% and 38.5%, respectively. Concurrently, this study found that essential medicines were poorly available in public health facilities and slightly had better availability in private sector and faith-based facilities as suggested by recent studies in Malawi [17] and Rwanda [18]. Despite the poor availability of medicines in Rwanda’s public sector, they were still affordable due to the fact that Rwanda has a wide coverage of health insurance. In general, this finding comparatively showed better access to medicines than the findings of studies done in many LMICs, including the current one in South Sudan.

Like the majority of African nations, South Sudan has inadequate access to medicines, defined by high prices, limited availability, and high costs as echoed by the finding of this study. WHO reported that between 50 and 60% of the African population lacks access to effective and high-quality medicines. A study done in eight sub-Saharan African countries showed that the availability of life-saving medications for women and children was unacceptably low [19]. Another finding in Cameroon and the Democratic Republic of Congo (DRC) reported that the average availability of antibiotics and non-communicable diseases (NCDs) medicines varied more between health facilities. The highest availability (70%) was scored by medicines against NCDs in Cameroonian church health facilities. LPGs at government and church health facilities were reasonably priced, and five of the seven antibiotics examined were inexpensive. In comparison, only one of the five NCD medications evaluated was cheap in each country. The originator brand medicines sold by private pharmacies were manifestly costly, and pharmaceutical costs in Cameroon were much higher than in the DRC. The availability of medicines was less than eighty per cent in a study done in Abuja, Nigeria, on cardiovascular, diabetic, and global medicines throughout various pharmaceutical sectors. High prices of medicines were also reported in the same study [20].

Contrary to the findings of the current research, A study in Ruweng state, South Sudan, found the average medicines availability of 83%, in public health facilities under direct donors’ supply. However, these findings do not accurately reflect the situation in South Sudan as the study was done in a sector heavily supplied by multi-donors and in one health sector [21].

Affordability of medicines is determined by the cost of a complete course of treatment in terms of days’ wages. Medicines are made affordable by making them available in the public sector, provision of public health insurance and regulating patient prices. South Sudan lacks public insurance coverage and has a weak pharmaceutical regulatory system that does not regulate patient prices. Despite the fact that medicines are provided free of charge in the public sector, a lack of availability forces people to purchase medicines in the private sector.

The findings of this study revealed that only two of the 29 medicines investigated were affordable. The other 27 medicines had higher median price ratios, making them unaffordable in private pharmacies, private clinics, and faith-based health facilities. NCDs needed more days' wages for a single course of treatment than the other types of medicines studied. In private clinics, amitriptyline, atorvastatin, and propranolol required 33.7-, 23-, and 22-day wages, respectively, for one complete course of treatment. The same three medicines required 20.2-, 23.6- and 16.9-day wages to buy one course of treatment in private pharmacies. As a result, medications used to treat NCDs were exceedingly unaffordable in South Sudan.

Many studies in LMICs reveal higher availability but inadequate affordability in the majority of cases [5]. The situation in South Sudan is compounded by the fact that essential medicines are scarce in the public sector and expensive in other sectors. This makes essential medicines extremely unaffordable.

## Limitations of the study

The study surveyed 29 medicines only instead of the 50 medicines recommended in the WHO/HAI methodology. This survey captured data at one point in time only, so it does not cover availability and price changes over time. Initially, it was intended to survey an equal number of facilities in all four sectors but only four faith-based facilities were surveyed. Finally, the study used the prices from the MSH international reference prices version 2015 to compute the Median Price Ratios which could not reflect the actual prices at the time of the study.

## Conclusions

The study showed that medicines were poorly available in all four surveyed sectors in South Sudan notably in the public sector, where the average availability was 50.4%. Most medicines were unaffordable in private pharmacies, private clinics and the faith-based sectors due to high prices. Only 0.07% of the surveyed medicines were affordable. Most of the surveyed medicines showed high price ratios with private pharmacies scoring an MPR of 4.64. Poor medicines availability in the public sector contributes to the overall unaffordability of medicines in all the other sectors. Therefore, there is low access to medicines in South Sudan. Hence, this study has provided evidence on prices, availability and affordability of medicines in Juba County, South Sudan. Based on these findings, policymakers may have some substantial evidence to formulate policies that will regulate prices, and increase availability and affordability of medicines. The findings can also be used as the basis to improve the public health system and re-organize the pharmaceutical sector in South Sudan to increase access to medicines. The generalization of the findings of this study not possible taking into consideration the potential changes in prices and availability of tracer medicines in remote locations from Juba County, especially in the Private Sector.

## Policy recommendations

Based on the findings of this study, we recommend:

- To increase medicine availability in the public sector.
- Adopt price regulation policies, especially in the private sector.
- Improve funding of health sector.

## Data Availability

Data sets and materials for information in this manuscript can be provided by the first author upon reasonable request.

## Abbreviations

BMZ: German Federal Ministry for Economic Cooperation and Development
DRC: Democratic Republic of Congo
EMF: Emergency Medicines Funds
HAI: Health Action International
HIV/AIDS: Human Immune Virus/Acquired Immunodeficiency Syndrome
HPF: Health Pooled Funds
IRPs: International referent prices
LMICs: Low-and-Middle income Countries.
LPGs: Lowest priced generics
LPGW: Lowest Paid Government Worker
MOH: Ministry of Health
MPR: Median Price Ratio
MSH: Management Sciences for Health
NCDs: Non-communicable diseases
NEML: National essential medicines list
PHCC: Primary health care centre
SDG: Sustainable Development Goal
SSEML: South Sudan Essential Medicines List
SSP: South Sudan Pound
TB: Tuberculosis
UHC: Universal Health Coverage
UN: United Nations
US: United State
USAID: United States Agency for International Development
WHO: World Health Organization
